# Comparative evaluation of the efficiency of segmental maxillary canine retraction using Burstone T-loops versus elastomeric chains: a split-mouth randomized clinical trial

**DOI:** 10.1590/2177-6709.30.2.e2524233.oar

**Published:** 2025-05-23

**Authors:** Mohammed Abd-Elaziz Abd-Elaziz YOUNES, Nehal Fouad ALBELASY, Ahmed Maher FOUDA

**Affiliations:** 1 Mansoura University, Faculty of Dentistry, Department of Orthodontics (Mansoura, Egypt).

**Keywords:** Segmental canine retraction, Power chains, Sliding technique, Frictionless retraction, Sectional T-loop, Retração segmentada do canino, Power Chains, Técnica de deslizamento, Retração sem atrito, T-loop segmentado

## Abstract

**Objective:**

The aim of this study was to evaluate and compare the effectiveness of elastomeric power chains and T-loop springs in retracting the maxillary canine with a time frame of four months.

**Material and Methods:**

The study included 16 patients (8 women and 8 men), with a mean age of 19.06±2.22 years. These patients were recommended to bilateral maxillary first premolars extraction. A split-mouth design was used to randomly assign opposing quadrants to either power chains or T-loops. Study models and panoramic radiographs were acquired before and after canines retraction. The rate of canine retraction, rotation, tipping (primary outcome), and molar anchorage loss (secondary outcome) were measured using study models and panoramic radiograph, after retraction. All data was subjected to appropriate statistical analysis.

**Results:**

The average retraction for T-loops was 4.76 ± 1.02 mm, while for power chains it was 4.28 ± 0.81 mm. T-loops showed faster canine retraction (1.19 ± 0.08 mm/month) than power chains (1.07 ± 0.06 mm/month). T-loops had a significantly higher canine rotation (17.28±2.62°) than power chains (10.62±4.15°) (*p*<0.005). The sliding side showed greater tipping than the loop side, with statistically significant difference. Anchorage loss was not statistically different between both mechanics.

**Conclusion:**

T-loops offered the highest degree of canine retraction with minimal tipping, while power chains used for sectional canine retraction resulted in less canine rotation. However, there was no significant difference in the amount of anchorage loss between both mechanics.

## RESUMO

**Objetivo:**

O objetivo deste estudo foi avaliar e comparar a eficácia de elásticos Power Chain e molas T-loop na retração do canino superior em um período de quatro meses.

**Material e Métodos:**

O estudo incluiu 16 pacientes (8 mulheres e 8 homens), com idade média de 19,06±2,22 anos. Os pacientes foram planejados para extração bilateral dos primeiros pré-molares superiores. Um design de boca dividida foi usado nesse estudo, para atribuir aleatoriamente quadrantes opostos aos Power Chain ou T-loops. Modelos de estudo e radiografias panorâmicas foram confeccionados antes e depois da retração dos caninos. A taxa de retração, a rotação e a inclinação do canino (desfecho primário) e a perda de ancoragem do molar (desfecho secundário) foram medidas, usando modelos de estudo e radiografias panorâmicas, após a retração. Todos os dados foram submetidos à análise estatística apropriada.

**Resultados:**

A retração média com as T-loops foi de 4,76 ± 1,02 mm, enquanto com os Power Chains foi de 4,28 ± 0,81 mm. As T-loops apresentaram retração mais rápida dos caninos (1,19 ± 0,08 mm/mês) do que os Power Chains (1,07 ± 0,06 mm/mês). As T-loops apresentaram rotação do canino significativamente maior (17,28 ± 2,62°) do que os Power Chains (10,62 ± 4,15°) (p < 0,005). O lado com Power Chains apresentou maior inclinação do que o lado com as T-loops, com diferença estatisticamente significativa. A perda de ancoragem não mostrou diferença estatisticamente significativa entre as duas mecânicas.

**Conclusão:**

As T-loops ofereceram a taxa mais alta de retração do canino, com inclinação mínima, enquanto os Power Chains usados para retração segmentada do canino resultaram em menor rotação do canino. Entretanto, não houve diferença significativa na quantidade de perda de ancoragem entre as duas mecânicas.

## INTRODUCTION

The goal of orthodontic treatment is to reposition teeth with maximum efficiency, while minimizing any negative impact on teeth and surrounding tissues. Canine retraction is the initial phase of the two-step technique for space closure.^1^ Various techniques for canine retraction are currently widely utilized, in which force can be exerted by means of various designs of elastics and closed coil springs. Conversely, frictionless mechanics involve the application of the sectional approach, such as Burstone’s T-loop, Rickett’s spring, or PG (Poul Gjessing) spring.

Pre-adjusted Edgewise mechanics for retraction uses the following two categories of principles: (i) the friction-based mechanics aims to distally slide the canine along a continuous archwire; (ii) the friction-less mechanics, which integrate loops (springs) into a continuous or segmented archwire, is designed to retract the tooth. There are two inherent drawbacks with any mechanics that uses a sliding mechanism instead of a simple tipping movement: friction and the magnitudes of forces cannot be readily determined, due to the relatively unknown and unpredictable nature of friction.^2^

The segmented arch mechanics recommends the division of the dental arch into three primary segments, particularly in cases involving extractions. These three segments consist of two posterior segments (teeth posterior to the extraction site) and one anterior segment (incisors and canines, in cases of first premolar extraction, which is the common tooth extraction pattern in Orthodontics).^3^

Segmented arch mechanics introduced two techniques for extraction spaces closure. The first technique involves canine retraction into the extraction spaces, followed by retracting the incisors or aligning the crowded incisors, in situations in which extraction was used to relieve the crowding of incisors or front teeth. The objective of this approach is to reduce the anterior displacement of posterior teeth, sometimes referred to as maximum anchorage.^3^ The second technique involve en masse retraction, in which all anterior teeth including the canines, are retracted into the first premolar extraction space as one unit. This method typically compromises anchorage, causing posterior teeth to move mesially.

Extensive studies have been conducted on the T-loop design for orthodontic space closure, with a specific focus on loops constructed with titanium-molybdenum alloy (TMA).^4^ The T-loop design typically ensures a consistent moment-to-force ratio (M:F), a uniform force intensity across the whole activation range of a closing loop, and a consistent low load-deflection rate.^4^

In response to the ongoing debate about the best mechanics for canine retraction, the present randomized clinical trial was conducted to evaluate the efficiency and suitability of Burstone T-loop (frictionless mechanics) and elastomeric power chain (friction mechanics) as segmented arch techniques for maxillary canine retraction. 

## MATERIAL AND METHODS

### STUDY DESIGN

This study was a randomized trial with a split-mouth design, with an allocation ratio of 1:1 between the two quadrants. There were no changes after trial initiation.

### REGISTRATION

This study was registered and approved by the Dental Research Ethics Committee of the faculty of dentistry at Mansoura University, Mansoura, Egypt (reference number: A01040230R) in April 2023. In addition, the trial (NCT05882526) has been registered on ClinicalTrials.gov. Enrollment of patients began in Jun 2023 and finished in March 2024. Every patient was included with information regarding the treatment procedures, and subsequently signed an informed consent. 

### SAMPLE SIZE, PARTICIPANTS, ELIGIBILITY CRITERIA, AND SETTING

#### Calculation of the sample size

The sample size for this study was determined using the software G*power v. 3.1.9.7 (Universität Düsseldorf, Düsseldorf, Germany). The calculation was based on the data reported from a previous study.^5^ The assumptions for the analysis were a paired t-test with a 95% power and a significance level of 0.05. Therefore, the determined sample size was 14 patients. The sample size has been increased by including 16 patients (32 canines), to deal with potential dropout during the study period. Thus, a total of 16 patients (8 men and 8 women) with an average age of 19.06 ± 2.22 years were selected from the outpatient clinic of the orthodontics department of Mansoura University School of Dentistry.

The following inclusion criteria were used: Class I malocclusion, with severe dental crowding or Class I bimaxillary dentoalveolar protrusion, age range of 17 to 23 years, fully erupted permanent canines, indication for both maxillary and mandibular first premolars extraction, no prior history of orthodontic treatment, good oral hygiene, and absence of any deleterious oral habits.

The exclusion criteria were as follows: poor oral hygiene, systemic diseases, preliminary signs of root resorption prior to orthodontic treatment, history of trauma or damage to the facial structures.

#### Randomization

The T-loops and power chains were allocated to either the right or left side, in a simple random manner, using random computer-generated numbers (Minitab v. 20 statistical software), with a 1:1 allocation ratio, by an academic staff member from the Department of Orthodontics, who was not involved in this research.

Identical opaque sealed envelopes, numbered in order, were used to conceal the allocation sequence, being opened only prior to the onset of maxillary canine retraction.

#### Blinding

The design of this study did not incorporate blinding for either the clinician or the patients. Nevertheless, the measurements (data collections) and statistical analysis phases involved blinding the researcher, to prevent bias in the investigation.

### INTERVENTIONS

One researcher treated all patients with a fixed, pre-adjusted Edgewise appliance (Roth prescription, 0.022 to 0.028-in slots) for the canines and second premolars, excluding bonding to the incisors.

**Figure 1:**
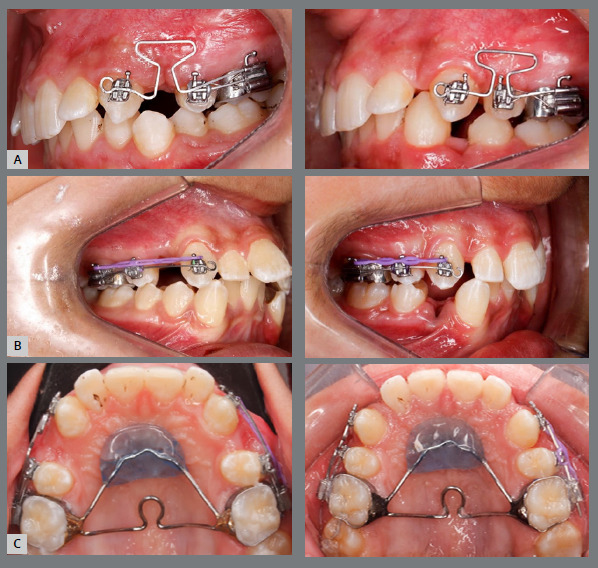
A) Intraoral photographs of a case with T-loop, before and after retraction. B) Intraoral photographs of a case with power chain, before and after retraction. C) Occlusal photographs of a case, before and after retraction.

Transpalatal arches with Nance buttons were utilized as anchorage for all patients, along with banding of the first molars using an Edgewise tube with a 0.022-in slot. The molars and premolars were secured together using a 0.016×0.022-in stainless steel wire, or they were anchored with stainless steel ligatures, or both methods were used simultaneously. Extraction of the first premolars was carried out in both arches without any prior leveling and alignment. Canine retraction was started at same time on both sides, using either power chains or T-loops (Fig. 1).

### BURSTONE T-LOOP SPRING

**Figure 2:**
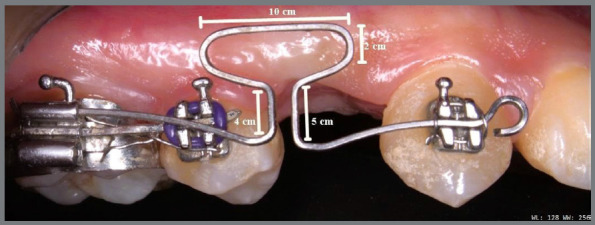
Design of the Burstone T-loop.

The T-loops were made from a 0.017 x 0.025-in TMA wire (Ormco Corp., Orange, Calif.) as segmented wire, with the standard dimensions described by Kuhlberg and Burstone^6^ (Fig. 2). Passive springs were adjusted in the molar auxiliary tube and the canine bracket. The anterior vertical arm, with 5-mm length, was placed in the bracket of the canine, and tied with ligature wire. Additionally, a 4-mm long posterior vertical arm was engaged into the first molar tube’s auxiliary slot, and was cinched back. The T-loop springs were positioned at the center of the extraction space. Pre-activation bends with 30° were incorporated into the T-loop at six different points, for a total pre-activation of 180°. To minimize disto-palatal canine rotation and adjust the inter-canine width during retraction, 35° anti-rotation bends were created in the anterior arms. By using a digital caliper with 0.01-mm accuracy (Mitutoyo, Sakado, Japan), a 3-mm activation was obtained. 

### SECTIONAL WIRE GUIDED RETRACTION USING POWER CHAIN

**Figure 3:**
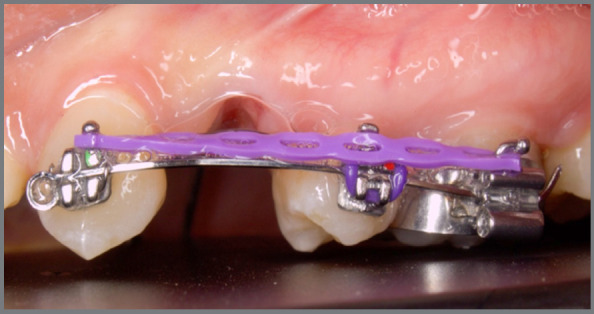
Sectional wire guided retraction using power chain.

The sectional wire on the contralateral side was made of 0.016 × 0.022-in stainless steel (Dentaurum, Ispringen, Germany). The sectional wire was placed in the first molar band, the second premolar and the canine. The canine was additionally fixed to the sectional wire with a ligature tie, to minimize rotation during movement. It was cut mesial to the canine with stop coil bend, to prevent dislodgement of the wire. Artistic bends were made to adapt the sectional wire to a passive fit, in cases of malpositioned teeth away from the distal sliding path of canines. A continuous (closed) elastomeric power chain was attached to the molar tube hook on one end, and was then stretched twice its original size, to generate 200 g of force, measured with a Dontrix gauge (American Orthodontics, Sheboygan, WI, USA), while the other end was attached to the canine hook (Fig. 3).

Control visits took place over a four-week period, to monitor canine retraction. During these follow-up appointments, the progress of tooth movement was assessed, the retraction spring was activated, and the power chain was replaced.

### OUTCOME ASSESSMENT

Outcomes evaluation was carried out using model analysis and radiographic assessment in panoramic radiographs. Rate of canine retraction, canine rotation, and mesiodistal axial inclination (tipping) were the primary outcomes measured. Changes in the position of the first molar (anchorage loss) were the secondary outcomes.

### MODEL ANALYSIS

Each patient performed two maxillary dental impressions: one immediately before canine retraction (T0), and another after the study period (T4), which lasted for four months. A Medit T710 scanner (Seoul, South Korea), known for its high accuracy (11 microns), was used to obtain digital models, by laser scanning plaster models.

The measurements were obtained using the 3Shape Analyzer computer software. The difference between the pre-retraction model (T0) and the model obtained after the four-month retraction period (T4) was used to calculate all measurements.

**Figure 4:**
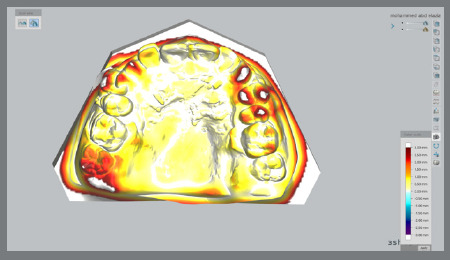
Color mapping of digitals models.

Digital models of the maxillary arch at T0 and T4 and superimposed models were used for the measurements. In the 3Shape software, the T0 and T4 models were chosen to carry out the superimpositions. An overlay of the two images was made possible by the selection of three stable reference points (palatal rugae region) from the T0 model, and their coordination with the reference points from the T4 model.^7^ Color mapping was used to confirm the accuracy of superimposition (Fig. 4).

### DETERMINATION OF CANINE RETRACTION RATE

**Figure 5:**
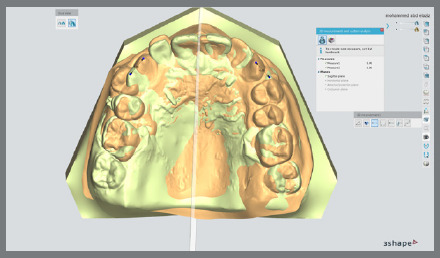
Superimposition of digitals models to determine canine movements.

The digital casts were superimposed using the medial aspect of the third rugae and the constructed midpalatal raphe, which was used as a reference median line for measurement and created by joining the anterior and posterior raphe points. Total canine retraction was measured at the cusp tip level (distance between two cusps) for both sides, from the cusp tip of the maxillary canine in T0 to the same point in T4 (Fig. 5). The rate of canine retraction was calculated by dividing the millimeter-measured amount of retraction by the four-month time interval.

### DETERMINATION OF CANINE ROTATION

**Figure 6:**
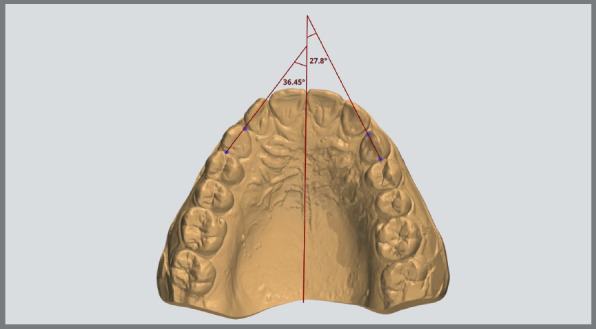
Determination of canine rotation on the digital model.

Lines were created by connecting the distal and mesial contact points of the canine. An angle was formed by the intersection of these lines and the midpalatal raphe (Fig. 6). The total rotation was measured as the difference between the values in the pre- and post-retraction.

### DETERMINATION OF ANCHORAGE LOSS

To measure the anteroposterior displacement of the maxillary first permanent molars, the occlusal distance between the identifiable point on the mesial marginal ridge at T0 and T4 on the superimposed models was measured. The maxillary molar displacement speed (mm/month) was then calculated. 

### DETERMINATION OF CANINE TIPPING

**Figure 7:**
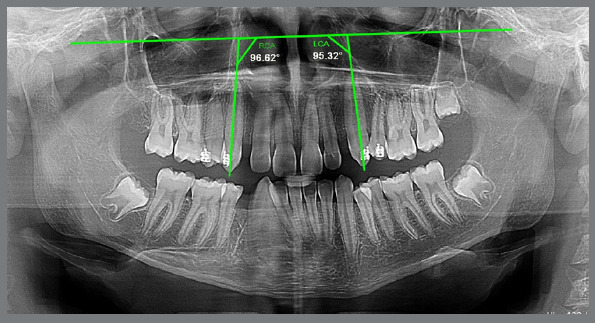
Determination of canine tipping after retraction, on panoramic radiographs.

The amount of canine tipping was measured on panoramic radiographs using the Micro Dicom viewer software (v. 2.0.0; Sofia, Bulgaria). The canine angulation was measured as the mesial angle created by the canine’s long axis on the infraorbital line. The infraorbital line was drawn at the most inferior points of the left and right orbits (Fig. 7). The difference between the degree of tipping in the pre-retraction and post-retraction panoramic radiographs was used to calculate the amount of canine tipping.

### METHOD ERROR

An analysis of the method error for the recorded measurements was conducted by randomly selecting ten casts one month after the initial measurement. The same software (3Shape) was used to repeat the superimposition, reestablish all reference points, and record all measurements.

A paired *t-*test was performed to assess procedural errors (investigation reliability). Furthermore, there was no statistically significant difference (*p*<0.05) between the two measurements. The intraexaminer reliability was also tested, using the intraclass correlation coefficient (Table 1).

**Table 1:** Intraclass correlation coefficient (ICC), showing the level of agreement.

Parameter	ICC
Retraction	0.994
Rotation	0.997
Anchorage loss	0.988
Tipping	0.990

### STATISTICAL ANALYSIS

The statistical analyses were conducted using the SPSS v. 25 statistical package (IBM Corp., Armonk, NY, 2017). The Shapiro-Wilk test was used to assess normality of data distribution. The results indicated that all measurements exhibited a normal distribution (parametric data), then, all outcomes were presented using descriptive statistics, and the data were presented as means and standard deviations. The treatment effects with T-loops and power chains were compared using a paired *t*-test. All tests in this study were conducted using a two-tailed approach, with a confidence level of 95%. A value of *p*<0.05 was set as the significance level.

## RESULTS

**Figure 8:**
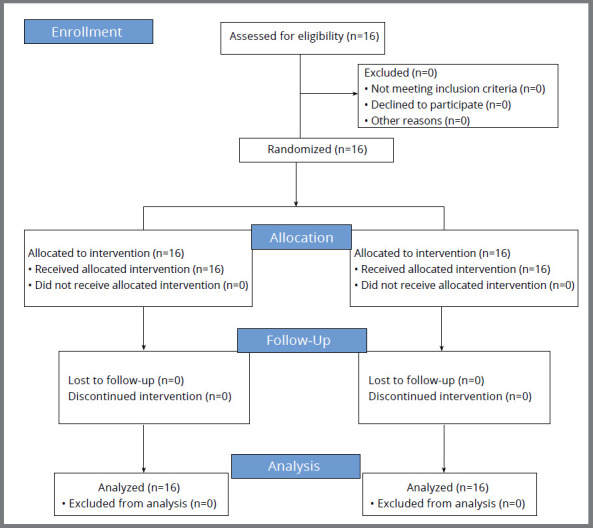
CONSORT diagram showing the flow of patients during the trial.

The study included 16 patients, with a mean age of 19.06 ± 2.22 years, being 8 women (50%) and 8 men (50%), as shown in Table 2. Figure 8 shows the CONSORT flow chart for clinical trial patient flow.

**Table 2:** Participant demographic data for the study.

Sex - n (%)	Age (years)	Mean ± SD
Male = 8 (50%)	18-22	18.88 ± 2.15
Female = 8 (50%)	17-23	19.25 ± 2.28
TOTAL = 16 (100%)	17-23	19.06 ± 2.22

The mean and standard deviation of total distal canine movement are shown in Table 3. After four months of canine retraction, the paired *t*-test result (p >0.05) showed no significant difference (4.76±1.02mm in T-loops and 4.28±0.81mm in power chains).

**Table 3:** Comparative analysis and descriptive statistics of changes from baseline to four months periods, in T-loops and power chain groups.

Outcomes	T-loop	Power chain	MD	95% CI		t	P value
	Mean ± SD	Mean ± SD		Lower	Upper		
Amount of retraction (mm)	4.76 ± 1.02	4.28 ± 0.81	0.48	-0.23	1.19	1.44	0.1715
Rate of retraction (mm/month)	1.19 ± 0.08	1.07 ± 0.06	0.12	0.07	0.18	4.64	0.0003*
Canine rotation (degrees)	17.28 ± 2.62	10.62 ± 4.15	6.67	4.29	9.04	6.37	<0.0001*
Anchorage loss (mm/month)	0.47 ± 0.39	0.39 ± 0.29	0.085	0.26	0.09	1.07	0.3029
Canine tipping (degrees)	7.50 ± 1.30	8.61 ± 1.34	-1.11	-2.07	-0.14	2.45	0.0271*

MD = mean difference; SD = standard deviation; CI = confidence interval, * Significant at p < 0.05, using paired t-test.

Compared to power chains (1.07±0.06mm/month), T-loops (1.19±0.08mm/month) had a higher rate of canine retraction, and the difference was statistically significant (*p* = 0.0003).

For T-loops, the distopalatal rotation of canine was17.28 ±2.62°, but for elastomeric chains, it was 10.62±4.15°. A statistically significant mean difference of 6.67° was observed (*p*<0.0001). However, power chains had significantly more canine tipping (8.61±1.34°) than T-loops (7.50±1.30°, *p* < 0.05). 

Over the four-month retraction period, T-loops and elastomeric chains recorded anchorage loss rates of 0.47±0.39 mm/month and 0.39 ±0.29 mm/month, respectively, although the difference was not significant (*p* = 0.4856).

## DISCUSSION

Canine retraction to the premolars extraction space is an inevitable phase in orthodontic treatment. To achieve the closure of these spaces in goal-oriented orthodontics, it is necessary to have a comprehensive understanding of the mechanical systems used. Either friction mechanics, which involves sliding, or frictionless mechanics, which uses loops, can accomplish space closure. Hence, clinicians consistently strive to identify and assess the superiority of one technique over another in retracting canines.

The current study used a split-mouth technique to standardize variables such as patient cooperation, oral hygiene, and bone thickness. Canine retraction started following extraction, to avoid any asymmetry between the two quadrants.

The selection of the elastomeric power chain for this study was based on the property of force degradation, which enables the uprighting of the canine during retraction. The nickel-titanium closed coil spring exerts a constant force for retraction,^8^ ^,^ ^9^ which was not favorable, due to its tendency to cause significant tipping of the canine and negatively impact the results. 

Reitan^10^ proposed applying 150 to 250g to ensure consistent movement of the maxillary canines. According to Brandt and Burstone,^11^ a force of 150 to 200g is considered reasonable for tipping canines. In the present study, the T-loop spring was activated by 3 mm every four weeks, in order to apply a force of approximately 150g, as the activation protocol suggested by Keng et al.^4^ According to the findings of Albelasy and Abdelnaby,^12^ no significant difference in canine retraction was observed when applying either 100g or 200g of force. In the sliding side, the elastomeric chain applied 200g during insertion, but, after 8 hours, it dropped to 100g and, after four weeks, to 40-50g, while maintaining the same length.^13^

The results revealed a significant difference (*p*=0.0003) in the rate of retraction of the canine between T-loops (1.19±0.20mm) and elastomeric chain (1.07±0.24mm). The higher rate of canine retraction using T-loops is corroborated by the results of Ziegler et al.^14^ and Hayashi et al.^15^ This can be due to the lack of binding and the application of a more constant force during the control visits by T-loops. On contrast, the lower amount of tooth movement on the side with sliding mechanics was most likely caused by mechanical rather than biologic factors. These mechanical factors include frictional binding and temporary or permanent stops in the movement of the teeth, caused by deformation and irregularities in the arch. 

The retraction rate measurements obtained using the T-loops in the present study were nearly in agreement with Mehta and Sable^16^ (about 1.3 mm/month), but were higher than those reported by Davis et al.^17^ (0.70 mm/month) and Keng et al.^4^ (0.80 mm/month). This disparities in values may be caused by the fact that both studies utilized different anatomic reference points or lines for variables evaluation.

Makhlouf et al.^5^ reported a low speed of canine movement in the T-loop side (with a mean difference of 0.1 mm per month), as the spring activation-preactivation procedure produced a high MF ratio and a minimal amount of retraction. In contrast to the findings of this study, the results of Nandan et al.^18^ indicated higher rate values (about 1.9 mm per month).This may be the result of a differences in distal activation of the spring, which was 6 mm, while in the current study was 3 mm.

The present study had a canine retraction rate of 1.07mm/month on the sliding side, which was approximately comparable to the rate mentioned by Hashemzadeh et al.^19^

Huffman and Way^20^ observed that Pletcher springs on 0.016” and 0.020” stainless steel wire retracted canines faster, with rates of 1.37mm/month and 1.20mm/month, respectively. Ziegler and Ingervall^14^ used an elastic chain on 0.018-in stainless steel wire to achieve retraction of 1.4mm/month in 3-4 weeks. This may be attributed to the use of smaller round cross-sectional wires. Bokas and Woods^21^ and Khanmasjedi et al.^22^ reported the highest elastomeric chain retraction rates (1.68mm and 1.89mm/month, respectively). Dixon et al.,^8^ Nightingale and Jones,^2^ and Chaudhari and Tarvade^23^ found the slowest canine retraction with the elastomeric chain (0.58, 0.84 and 0.3mm/month, respectively).

In terms of rotation, the maxillary canines showed a significant distolingual rotation with the loops technique (17.28±2.62°), which was lower than Nandan et al.^18^ and higher than Davis et al.^17^ and Masaes et al.^24^ On the other hand, the degree of tipping on the sliding side (10.62±4.15°) was higher than in the study of Hashemzadeh et al.^19^ It has been found that the retraction spring is not more effective than sliding mechanics in controlling canine rotation. There are multiple potential justifications for this outcome. In this study, the rotation in the loops was caused by a decrease in the angle of anti-rotation bends, which was 35°, as recommended by Burstone, and this is in contrast to the 90° anti-rotation bends proposed by Marcotte^25^ and the increased anti-rotation bends recommended by Nanda.^26^

Regarding tipping, the comparison of the tipping degree during canine movement in the present study favors the retraction spring, in approximately 7.5° distal tipping, as compared with approximately 8.61° with the sliding mechanics, and this coincides with Ziegler et al^14^ and Hayashi et al^15^ studies. The degree of tipping with the sliding mechanics would probably have been smaller if a stiffer archwire had been used. The more flexible wires will deflect, allowing more distal tipping, compared to stiff wires. Also, when force is applied away from the center of resistance during tooth movement, it leads to undesired tipping and rotation.

The tipping on the T-loops side was lower than that reported by Nandan et al.^18^ and Masaes et al.,^24^ higher than that of Davis et al.^17^ and Keng et al.^4^, and almost equivalent to the findings of Mehta and Sable^16^. On the elastomeric chain side, two publications^19^ ^,^ ^27^ reported lower tipping values than those found in the current study.

The study of Gandini et al.^28^ indicates that individual canine retraction resulted in anchorage loss when no anchorage was intended or utilized. The planned anchorage in the current study was achieved through the use of a transpalatal arch with acrylic buttons, which enhances anchorage strength. The difference in anchorage loss between the two mechanics was found to be insignificant (*p*=0.30), which can be related to the strong control exerted on the first molars. Loop mechanic (0.47mm/month) was smaller than the one reported by Nandan et al.^18^, and higher than Davis et al.^17^ However, sliding mechanics (0.39 mm/month) was similar to the one reported by Hashemzadeh et al.^19^

## CONCLUSIONS

This four-month study resulted in the following conclusions:

» Both mechanics, the power chain and the T-loop, are effective force delivery systems for maxillary canine retraction.
» T-loop mechanics (4.76 mm) resulted in a relatively higher rate of maxillary canine retraction than the sliding mechanics (4.28mm).
» T-loop demonstrated 7.50° tipping, indicating better control than the 0.016×0.022-in stainless steel sectional wire with power chain, which presented 8.61° tipping.
» The power chain side (10.62°) exhibited superior rotational control, compared to the T-loop (17.28°).
» During sectional canine retraction, there was no statistically or clinically significant difference in the anchorage loss rate between the two mechanics.
